# Cell division in *Escherichia coli *cultures monitored at single cell resolution

**DOI:** 10.1186/1471-2180-8-68

**Published:** 2008-04-23

**Authors:** Johanna Roostalu, Arvi Jõers, Hannes Luidalepp, Niilo Kaldalu, Tanel Tenson

**Affiliations:** 1 Institute of Technology, University of Tartu, Tartu, Estonia; 2 Zentrum für Molekulare Biologie der Universität Heidelberg, Heidelberg, Germany; 3 Quattromed Cell Factory, Nooruse 9, Tartu, Estonia

## Abstract

**Background:**

A fundamental characteristic of cells is the ability to divide. To date, most parameters of bacterial cultures, including cell division, have been measured as cell population averages, assuming that all bacteria divide at a uniform rate.

**Results:**

We monitored the division of individual cells in *Escherichia coli *cultures during different growth phases. Our experiments are based on the dilution of green fluorescent protein (GFP) upon cell division, monitored by flow cytometry. The results show that the vast majority of *E. coli *cells in exponentially growing cultures divided uniformly. In cultures that had been in stationary phase up to four days, no cell division was observed. However, upon dilution of stationary phase culture into fresh medium, two subpopulations of cells emerged: one that started dividing and another that did not. These populations were detectable by GFP dilution and displayed different side scatter parameters in flow cytometry. Further analysis showed that bacteria in the non-growing subpopulation were not dead, neither was the difference in growth capacity reducible to differences in stationary phase-specific gene expression since we observed uniform expression of several stress-related promoters. The presence of non-growing persisters, temporarily dormant bacteria that are tolerant to antibiotics, has previously been described within growing bacterial populations. Using the GFP dilution method combined with cell sorting, we showed that ampicillin lyses growing bacteria while non-growing bacteria retain viability and that some of them restart growth after the ampicillin is removed. Thus, our method enables persisters to be monitored even in liquid cultures of wild type strains in which persister formation has low frequency.

**Conclusion:**

In principle, the approaches developed here could be used to detect differences in cell division in response to different environmental conditions and in cultures of unicellular organisms other than *E. coli*.

## Background

The ability to grow and divide is common to all forms of life and it is the most widely studied aspect of bacterial physiology. This parameter has great importance from both medical and scientific points of view. Common methods for investigating bacterial cell division and growth include monitoring the cell's ability to form colonies on solid media and evaluating the increase in cell number over time in a liquid medium as a function of the turbidity of the culture. These methods are fast and cheap and provide sufficient data to evaluate growth and division at the level of the whole bacterial population. However, it has emerged in recent years that genetically homogeneous bacterial populations are often physiologically heterogeneous and contain several subsets of cells with different properties [1-3]. The phenotypic heterogeneity may be caused by noise or bi-stability of gene expression [4]. Differences at the level of gene expression may also translate into more complex physiological phenotypes. Formation of endospores of bacilli and fruiting bodies of *Myxococcus *are familiar examples of such differentiation. More cases of epigenetic heterogeneity in *Bacillus subtilis*, such as swimming or chaining of vegetative cells, cannibalism upon entry into sporulation and development of genetic competence, have been traced recently to heterogeneity in the expression of certain regulators [2].

Another example of phenotypic heterogeneity is the formation of persisters [5]. These are non-growing or slowly-growing bacteria that are tolerant to the bactericidal activity of antibiotics. After removal of the antibiotic, persisters resume growth and thus may be responsible for the survival of an infecting population when antimicrobial therapy is discontinued. Persisters can be found in small numbers within exponentially growing bacterial cultures, whereas the frequency of tolerant bacteria increases upon entry into stationary phase and during biofilm formation [6,7].

An additional source of heterogeneity during prolonged stasis is accumulation of damage and mutations [8]. Non-growing cells gradually lose their ability to form colonies but many of them may retain membrane potential and are described as viable but nonculturable (VBNC) [9]. Whether nondividing subpopulations consist of deteriorated and prospectively dying cells or specialized survival forms is a matter of contention [9,10]. All of this adds to the levels of heterogeneity in bacterial populations and makes observation of the growth rates of individual bacteria interesting.

Whereas counting the colony forming units (CFU) or measuring the turbidity of the culture fails to take heterogeneity into consideration, microscopy, specifically live-cell imaging, overcomes this problem. It enables individual cell divisions to be followed and different subpopulations in the growing culture to be distinguished [11,12]. However, live-cell imaging is often time-consuming and image analysis can be subjective, unless custom-made software is used. In addition, the number of cells analyzed per experiment is small and often not sufficient for statistical analysis, let alone isolation of cells from specific subsets for further experimental procedures.

Flow cytometry is used for measurements at the single cell resolution and interesting subpopulations can be isolated using fluorescence activated cell sorting. Here we develop a novel flow cytometric assay that enables cell division to be followed in liquid cultures at the single cell resolution. Using our assay, we show that *E. coli *cells divide homogenously during exponential growth phase and also while entering stationary phase. In stationary phase, growth and cell division cease uniformly across the whole population. However, while recovering from stationary phase, the population splits into two subsets, one of which is able to recover from stationary phase and resume growth whereas the other is not. In contrast to the dividing subset, the non-dividing cells are able to survive ampicillin treatment and start growing after the antibiotic is removed from the environment.

## Results

### Flow cytometry-based assay for monitoring individual cell divisions in *E. coli *cultures

**Figure 1:**
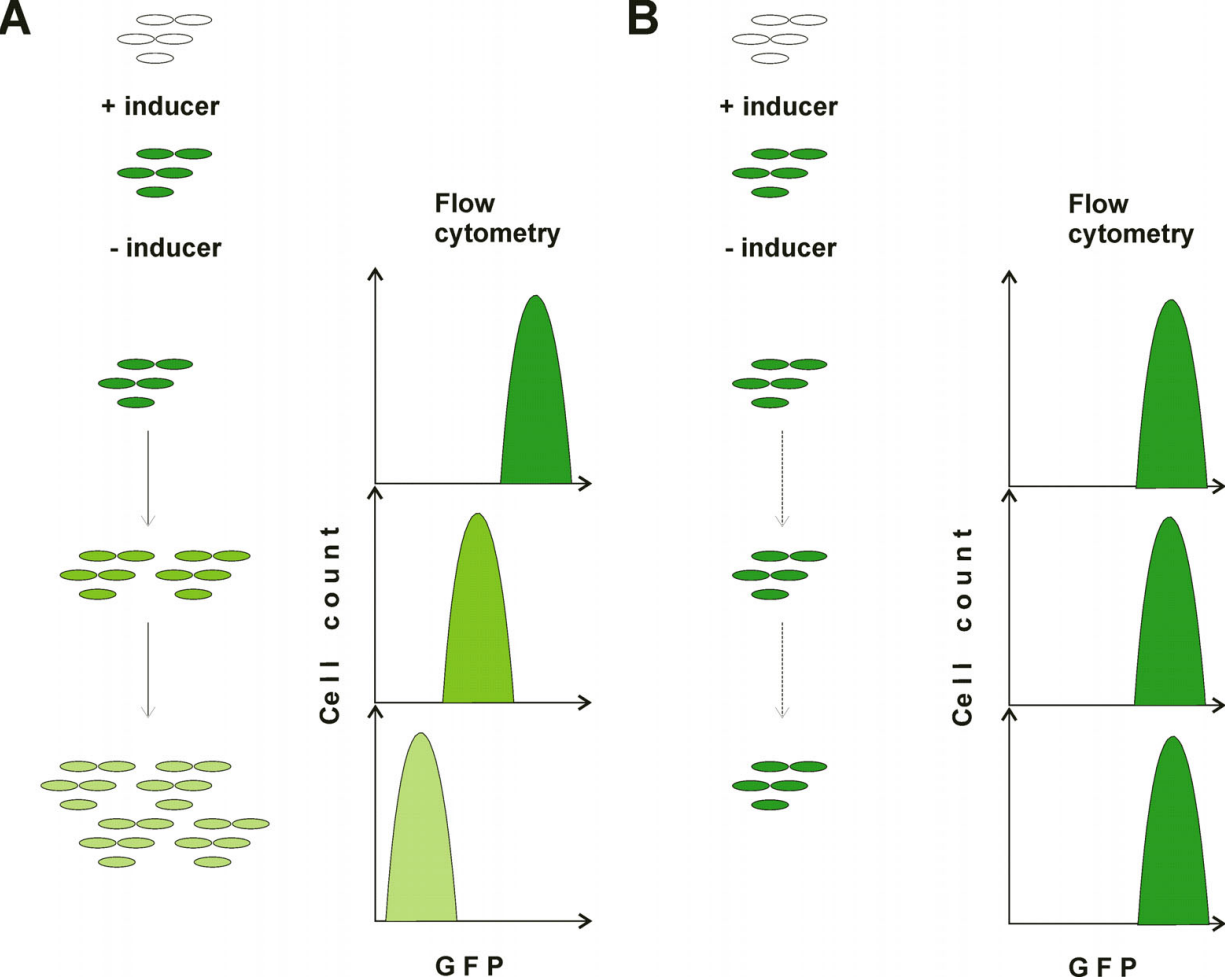
**Schematic representation of the GFP dilution experiment.** Expression of GFP is induced with HSL. After the cells have accumulated GFP the inducer is removed by collecting the cells by centrifugation and placing in fresh (or conditioned) medium. In growing cells (A) the amount of GFP decreases with each cell division. In non-growing cells (B) the amount of GFP remains constant.

**Figure 2:**
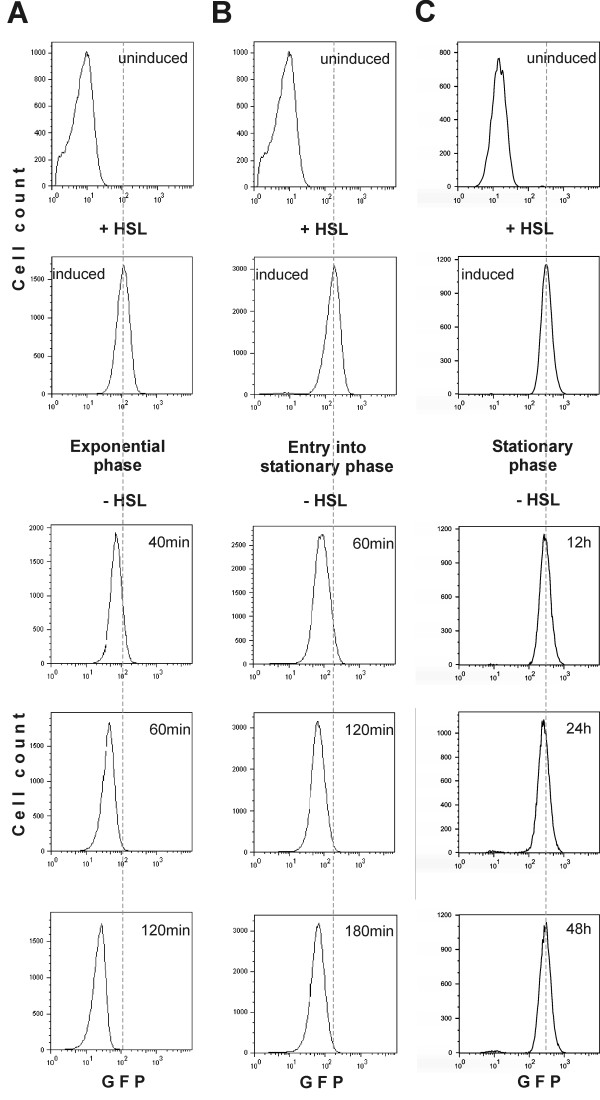
The GFP dilution experiment shown in Figure 1 as performed on exponentially growing (A), early stationary phase (B) and stationary phase (C) cultures.

The presence of heterogeneity in several bacterial populations [1,2] led us to ask whether isogenic *E. coli *cells have equal capacity to divide and produce progeny. In order to answer that question we developed a flow cytometry-based assay that enables individual cell division events in the population to be tracked (Figure 1). Cells were labelled by inducing the expression of green fluorescent protein (GFP). GFP was expressed from a promoter inducible by LuxR protein when a homoserine lactone (HSL) supplement was provided as inducer. *E. coli *is not able to synthesize HSL but has a specific HSL receptor [13]. Therefore, HSL by itself might induce physiological changes. To investigate this issue, IPTG inducible *tac *promoter was used for GFP expression [14], giving very similar results to the HSL-inducible system in experiments similar to those shown in Figure 2 (Additional file 1; data not shown). We conclude that the HSL induced physiological changes are not significant in the context of current study. This suggests that, in principle more complex reporter systems can be built by combining HSL and IPTG inducible promoters with the fluorescent proteins of different colours.

After verifying by flow cytometry that the cells do indeed express GFP, the bacteria were transferred into medium without inducer and changes in GFP signal intensity were monitored over time. We used a highly stable isoform of GFP, GFPmut2 [15], which was not degraded during the timeframe of our experiments (the amount of GFP does not decrease during incubation of cells treated with chloramphenicol, an inhibitor of protein synthesis; data not shown). Therefore, the stepwise weakening of the GFP signal intensity served as a good indication of cell divisions since it could only result from reduction of the number of GFP molecules per single cell, which in turn could only happen when cells divided. On a histogram of fluorescence intensity, a population in which all the cells are propagating at an equal rate would form a single peak of GFP fluorescence that decreases in intensity over time (Figure 1). Alternatively, a population that consisted, for example, of dividing and non-dividing cells would generate two peaks of GFP fluorescence. One peak would show a decrease in GFP intensity and correspond to the dividing cells, and the other, representing the non-dividing subset, would retain its high fluorescence. The use of similar GFP expression systems in studies on protein diffusion has revealed that this protein accumulates in large amounts [16-18], evading the high stochastic noise that is observed when very few molecules are present [4]. GFP is also a soluble protein, freely diffusing in the cytoplasm [16-18]. Therefore our experimental approach is expected to detect cell division rather than effects created by stochastic dilution processes or protein aggregation.

### *E. coli *exponential phase cultures consist of uniformly dividing cells whereas stationary phase cultures contain uniformly non-dividing cells

First we analyzed cell divisions in an exponentially growing *E. coli *MG1655 population (Figure 2A). Cells of the growing culture were treated as described above. GFP expression was induced with homoserine lactone (HSL) for 1 hour; the cells were subsequently released into fresh medium devoid of the inducer. At the indicated time points cells were harvested for flow cytometry, which revealed a homogeneous population (i.e. a single fluorescence peak, labelled by a dotted line in Figure 2) and continuous reduction in GFP signal intensity over time across the entire population of cells. The rate of GFP dilution as estimated from the 40 minute and 60 minute time points (Figure 2A) corresponds to the doubling time of the cells (approximately 25 min). This shows that in exponential growth phase, the vast majority of *E. coli *cells divide at similar rates. We observed that after 60 minutes the GFP signal is diluted to values only slightly increased over the background level and therefore a quantitative relationship between growth rate of the culture and GFP dilution values is lost. Still, in these longer time points all cells behave in uniform manner.

It is generally assumed that bacterial populations in natural habitats either grow very slowly or do not proliferate at all owing to the low availability of nutrients. This state is comparable to the stationary phase of a batch culture. In the literature, the stationary phase is characterized as a stage in which cell division is halted. This is inferred from the fact that the number of colony forming-units per millilitre as well as the cell number measured by microscopy in stationary phase *E. coli *cultures remain unchanged for several days [19,20]. However, lack of cell division is only one possible interpretation of this observation. Colony forming capacity would also remain on a plateau in a dynamic bacterial population in which cells are dividing and dying at similar rates. There was no direct experimental evidence to confirm either of these hypotheses. Also it is unknown whether all cells stop growing at the same time upon entry into stationary phase. Our assay enabled us to address these questions and the results favour uniform slowdown and quiescent subsistence in the stationary phase. To investigate entry into stationary phase, cells were pulse-labelled with GFP and then grown until stationary phase was reached. As shown in Figure 2B, all bacteria gradually slow down until they eventually cease to divide and enter a non-proliferating state at the same time (between the 120 and 180 min time points when the GFP signal shows no further weakening). A similar experiment was carried out to test cell proliferation in deeper stationary phase. The GFP signal did not decrease in intensity and remained at the level of the initial induction even after the cells had spent two days in stationary phase and the culture remained homogenous (Figure 2C). In some experiments a small population of cells (less than 1%) started to dilute out GFP. The nature of this cell population and the reasons behind its irreproducible occurrence remain to be determined. In general, our results show that bacteria in stationary phase do not divide and remain quiescent. This conclusion is valid for the bacterium *E. coli *growing in a particular medium for certain time periods. It remains to be seen if cell division in the stationary phase is observed when any of the parameters is changed. The methods developed in the current study allow investigating this issue.

### Upon recovery from stationary phase *E. coli *culture differentiates into dividing and non-dividing subpopulations of cells

**Figure 3:**
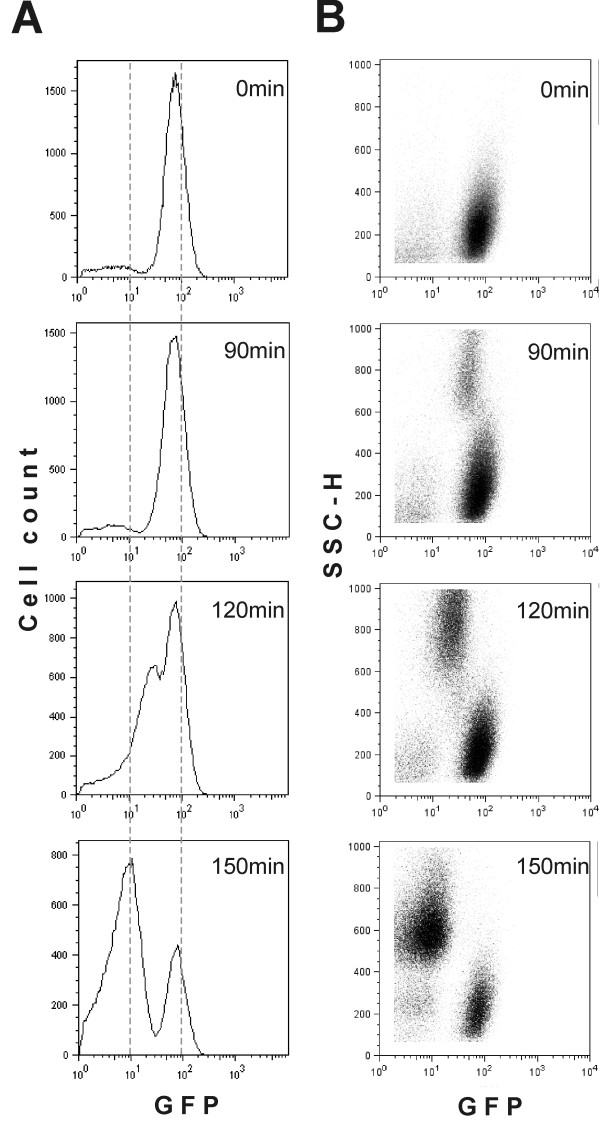
**The GFP dilution experiment described in Figure 1 as performed on cultures freshly inoculated with stationary phase cells.** Changes in the amount of GFP per cell are shown in panel A and the correlation between the amount of GFP and the side scatter parameter (SSC-H) is shown in panel B. As a fixed number of events (30,000) was counted per sample, the relative distribution but not the peak size can be compared between different panels.

It should be beneficial for a single bacterial cell to start dividing quickly whenever the conditions favour growth, thereby propagating its genetic material as efficiently as possible. In order to imitate this situation, we followed the ability of cells from an *E. coli *batch culture to recover from stationary phase and start dividing. Cells expressing GFP were kept in stationary phase for 24 hours and then diluted in fresh medium to allow growth. The emergence of two peaks of GFP fluorescence in the recovering population indicates that only a subset of cells is able to start reproducing (Figure 3A). Only a fraction of the initial population showed a gradual reduction in GFP signal intensity when nutrient became available. The rest retained their high GFP fluorescence and were therefore not dividing.

In addition to detecting fluorescence, standard flow cytometry equipment also measures light scattering by the particles. The "forward scatter" is defined as the scattering that occurs between the light source and a detector positioned in line. This parameter usually correlates with particle size [21-23]. Another detector is positioned at 90° to the light source and measures the "side scatter" (SSC-H) parameter. In eukaryotic cells, SSC-H is descriptive of intracellular granularity [23]. In bacteria, SSC-H has been argued to reflect cell size, but the possibility that it shows the properties of the cell wall, ribosome content or amount of macromolecules per cell instead cannot be excluded [23]. Curiously, we found that SSC-H enables dividing and non-dividing bacteria to be distinguished: the SSC-H value was considerably higher in dividing than in non-dividing cells (Figure 3B). During recovery from stationary phase, the increase in SSC-H always preceded the reduction of GFP signal intensity and first cell division. Therefore, although we cannot explain the reason for changes in SSC-H, it provided us with a tool to distinguish between future dividing and non-dividing cells even before actual cell division occurred. This allowed us to estimate the size of the cell population that starts growing by following the number of particles using the increase in the SSC-H parameter. Transition into the growing state is taking place as a single "burst" approximately 90 minutes after inoculation. We estimate that in our experiments about one third of cells resume growth in this initial "burst" while two thirds of the inoculum remains nongrowing. During further incubation the recovered subpopulation is growing exponentially with an approximate doubling time of 30 minutes. The lack of bacteria with intermediate fluorescence shows that cells of the initially nongrowing subpopulation do not resume growth later and stay nondividing.

**Figure 4:**
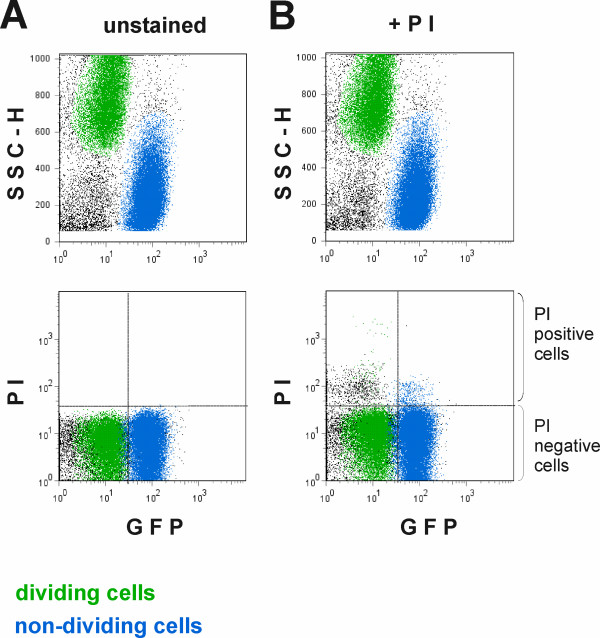
**Staining of the cultures recovering from stationary phase with propidium iodide, a probe that stains only the cells that have lost membrane integrity.** In the upper dot blots both the unstained control population (panel A) and the PI treated population (panel B) were classified into dividing (green) and non-dividing (blue) cells on the basis of GFP signal intensity and SSC-H value (as in Figure 3). In the lower dot plots the same subpopulations were then plotted on a GFP vs. SSC-H graph to determine the number of PI-stained dead cells they contained. PI treatment (panel B, below) resulted in very few PI-stained cells in both the dividing and non-dividing subpopulation compared to the unstained control (panel A, below).

We considered the possibility that the subset of the population unable to recover from stationary phase might consist mainly of dead cells. The culture that contained dividing and nondividing cells was treated with propidium iodide (PI), a membrane-impermeable stain that does not enter intact (living) cells but stains bacteria that have lost their membrane integrity [24]. Staining with PI is often used to differentiate dead bacteria from living cells [24]. It appeared that the cells in both the non-dividing and the dividing subsets retained their membrane integrity since only a few in each subset were stained with PI (Figure 4). We conclude that the differentiation into dividing and non-dividing subsets upon recovery from stationary phase does not result from massive cell death in the population.

**Figure 5:**
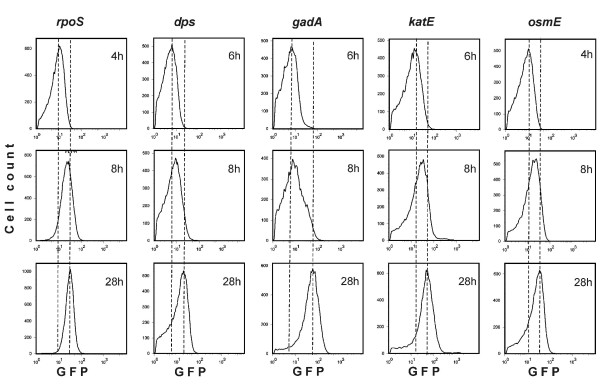
**Expression of stationary phase-specific genes.** The promoters and translation initiation regions were inserted in front of the ORF coding for GFP. The experiment was started (zero time) by inoculating a fresh culture. The samples were taken at times indicated. The dashed lines, added for alignment purposes indicate the location of GFP peak in the beginning and end of the experiment.

Another possible difference between the two subsets may lie in a differential ability to respond to the stress conditions that cells face in stationary phase. It is conceivable that the subset of bacteria unable to start reproduction upon dilution with fresh medium might be defective in some of the stress response pathways. To address this hypothesis, promoter regions of some stationary phase-specific stress response genes [25,26] were cloned in front of the GFP encoding sequence on a low copy number plasmid. GFP expression was monitored when cells started progressing from log phase (4–6 h) to stationary phase (8–28 h) (Figure 5). Note that in these experiments flow cytometry was used to follow the induction of *de novo *GFP expression, not GFP dilution as in the experiments described earlier. This analysis revealed slightly different expression kinetics of these constructs: expression from the *rpoS*, *katE *and *osmE *promoters was already visible in early stationary phase (8 h), whereas GFP expressed from the *dps *and *gadA *promoters only started accumulating later (28 h) (Figure 5). Data were also obtained at 48 h, 72 h and 96 h and gradual increase in the expression of all test constructs was observed (data not shown). Surprisingly, however, at least in deeper stationary phase (28 h and later), expression from all these promoters seemed to be relatively homogeneous throughout the population and certainly did not show a binary pattern. A construct containing the GFP coding gene under the HSL dependent promoter was introduced without adding the inducer. This control plasmid shows no expression in the stationary phase (Additional file 2). Thus, the occurrence of dividing and non-dividing subsets cannot be attributed to differential activation of stress response pathways in individual cells in stationary phase cultures.

### The nongrowing subpopulation tolerates ampicillin treatment and in part starts dividing after removal of the antibiotic from the growth medium

**Figure 6:**
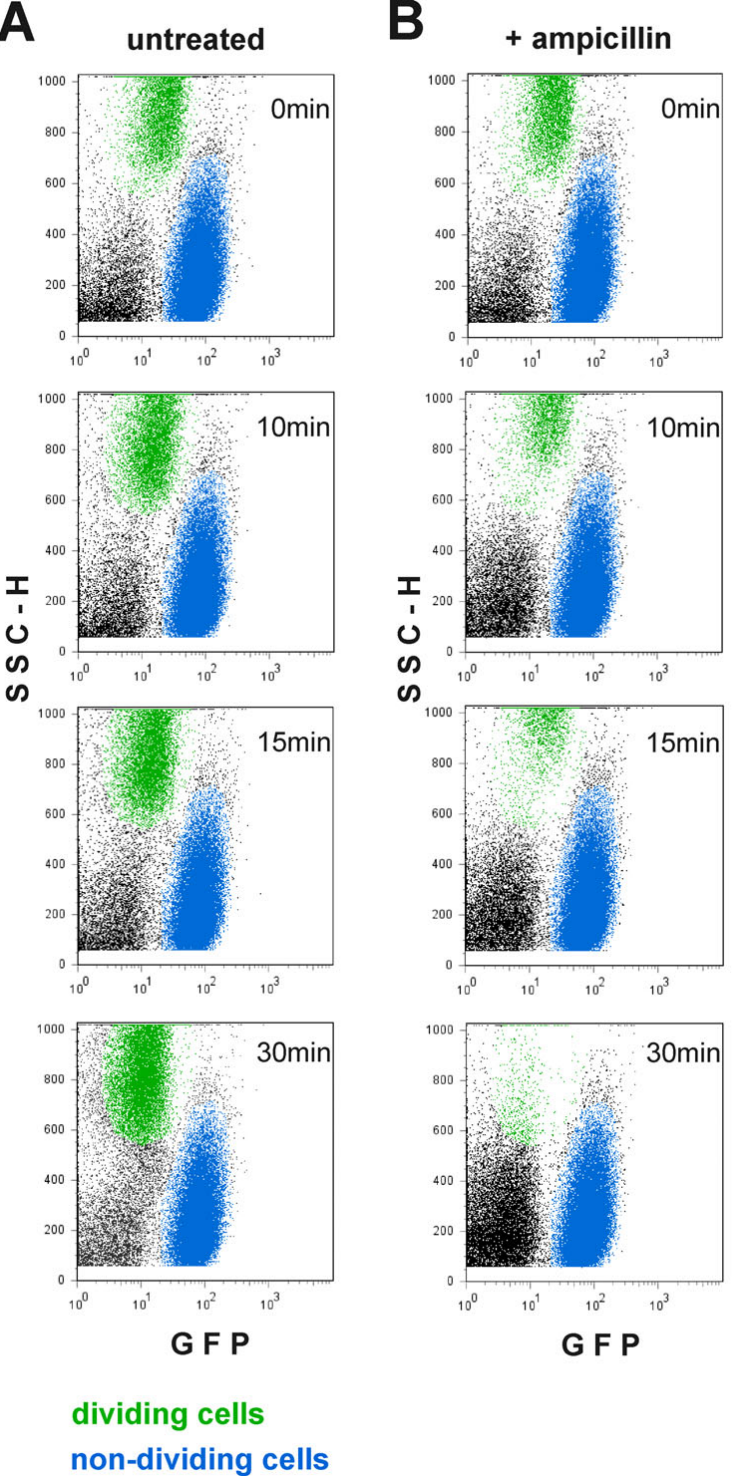
**Lysis of the growing cells with ampicillin.** The stationary phase cells containing GFP were diluted into fresh medium. After 2 hours of growth, ampicillin was added (zero time) and samples were taken at times indicated (B). An untreated culture was used as a control (A). The samples where analyzed by flow cytometry. Growing cells (high side scatter and low amount of GFP) are indicated in green, nongrowing cells (low side scatter and high amount of GFP) are indicated in blue, a population of debris particles in the flow buffer is indicated in black.

The fact that some cells resume growth rapidly and others remain dormant has important consequences for a microbial population. It is known that several stress factors, including bactericidal antibiotics, act only on actively growing cells; bacteria that are not growing are refractory to antibiotics [27]. Persisters are tolerant to killing because they are temporarily non-growing but are able to switch back to the growth mode later when the hazard has passed [11]. We tested whether the non-dividing subpopulation we observed contains persisters. To fulfil persister criteria, the cells should firstly survive incubation with a bactericidal antibiotic and secondly recover and start to grow later. We exposed the culture containing dividing and non-dividing cells to ampicillin, the drug that has been used previously for monitoring and isolation of persisters from mutant *E. coli *strains that form persisters at abnormally high frequency [6,11]. As expected, ampicillin lysed the growing cells within 30 minutes but did not affect the non-growing population (Figure 6). Quantification of the ampicillin lysis is provided in Additional file 3.

**Figure 7:**
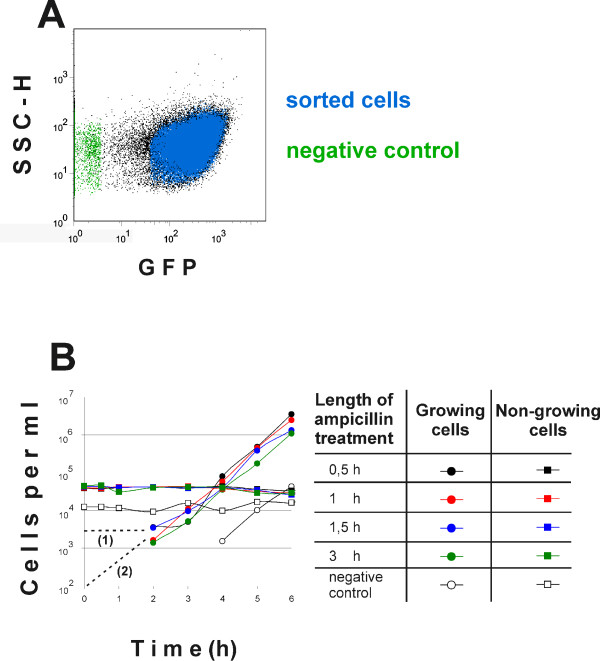
**At least some of the cells that survive ampicillin treatment are able to resume growth.** The cells were treated with ampicillin as described in Figure 6. The cells not lysed by ampicillin were isolated by flow cytometric sorting (A, blue). A population of debris particles (A, green) was used as a control for sterility of the procedure. For some counting events it was impossible to decide if they represent cells or debris particles. These events are shown in black. The cells were treated with ampicillin for 0.5, 1, 1.5 or 3 hours, isolated and inoculated into fresh medium. Increase in the numbers of growing and nongrowing cells was followed by flow cytometry (B). The growing and nongrowing cells were defined by their GFP content and SSC-H parameters as described in Figure 3. Extrapolations for estimating a minimum (2) or maximum (1) percentage of persisters are indicated by dashed lines.

To test whether the non-lysed cells are persisters or can no longer be cultured, we collected the ampicillin-treated, non-growing population by flow sorting and allowed it to recover in a fresh medium devoid of ampicillin. As a control, we flow-sorted and transferred into growth medium an equal number of non-fluorescent particles, aggregates in the sorting solution that are not living *E. coli *cells. In 3–4 hours we observed a new growing population of cells (Figure 7). The negative control remained sterile (or contained a very small number of contaminating cells) confirming that the growing bacteria indeed emerged from the non-growing pool and were not a result of contamination. To follow the fate of the bulk of the sorted non-dividing bacteria during incubation in the growth medium we counted the absolute numbers of cells in our samples. During 6 hours of incubation, the number of bacteria that retained high GFP contents and low SSC-H did not decrease significantly. It was not possible to continue the experiment further, since it became increasingly difficult to count reliably the non-dividing cells because of the rapidly increasing mass of growing bacteria (Figure 7). The doubling time of the growing population (Figure 7) was 25.0 ± 3.7 minutes, close to the doubling time of 24.2 ± 2.3 minutes measured for the exponentially growing culture in the medium used in the experiment. The incubation time with ampicillin had no particular effect on the recovery of growth, indicating that cells that are refractory to ampicillin are not damaged by longer exposure to the drug. Thus, combining our GFP dilution method with flow sorting, we were able to observe persisters in a culture of wild type *E. coli*.

If we assume that cells started to proliferate immediately after transfer into fresh medium and doubled in about 30 minutes, then approximately 100 cells per ml started dividing in this experiment. This constitutes approximately 0.2% of the sorted cells, giving us a minimum estimate of persistor frequency within the nongrowing population (Figure 7, dashed line (2)). The maximum estimate for persister frequency can be obtained from the presumption that all cells started growth immediately before the subpopulation of dividing cells was detected (Figure 7, dashed line (1)). This extrapolation indicates that the persisters cannot constitute more than 10% of the sorted cells.

## Discussion

Evaluating the rate of cell division by measurements based on a culture's ability to form colonies or on changes in cell turbidity overlooks the heterogeneity of bacterial populations. Cells can differ in age, levels of gene expression, damage suffered, etc. This, in turn, may result in differences in individual growth rates, which would remain undetected by such methods. For example, it has been shown that exponential phase cultures of both *Caulobacter crescentus *and *B. subtilis *consist of fairly different cell types: sessile cells and motile cell doublets or single cells [28,29].

Until recently, microscopy has been the sole technique for monitoring division of individual cells within the population. The benefit of the technique is that it allows to follow each single cell and its progeny through experiment [11,12]. However, there are several limitations to microscopy. Most notably, the number of cells analyzed per experiment is usually quite low, a few hundred at best, so events occurring at a low frequency might go unnoticed. In addition, changing the growth conditions is difficult and requires specialized equipment (flow cells) [11]. Also, analysis of microscopic data is tedious, time-consuming and sometimes subjective. We have developed a new method for monitoring cell division at the single cell resolution. The method is based on dilution of GFP upon cell division, monitored by flow cytometry (Figure 1). It enables large numbers of cells to be analyzed rapidly and the population's response to changes in environment to be monitored, and importantly, it can be applied to a liquid culture and in any laboratory with access to a flow cytometer. Different species of bacteria (and other unicellular organisms such as yeast) could potentially vary in degrees of heterogeneity with respect to cell division in different environments and stages of their life. Our method could be effectively applied to other microbes and used to study how differences in cell division correlate with other parameters such as cell size and levels of gene expression.

The fact that CFU counts, cell counts and culture turbidity remain unchanged for several days in stationary phase might indicate that there is no growth during stationary phase. It is still conceivable that stationary phase is a mixture of several subsets of cells, some actively growing and other dying. In this case, the respective growth and death rates should be numerically equal. Our results show that *E. coli *cells in culture divide uniformly in exponential phase (Figure 2). This result is in apparent contradiction with the previous report that *E. coli *cells containing an aged pole divide slightly slower than the cells with new poles [12]. As the reported differences in growth rate are relatively small and the cells containing an aged pole constitute a small fraction of the population [12], the resulting heterogeneity would be not detected by our flow cytometry based method.

When nutrient supplies approach exhaustion, cell division slows down homogeneously and division stops completely in stationary phase (Figure 2). Prolonged stasis (incubation times of more than 5 days) is known to be accompanied by increases in mutation frequency [30]. One physiological outcome of these stress-induced genetic alterations is the GASP phenotype (*growth advantage in stationary phase*) [31,32]. We did not monitor these later stages of stationary phase in the current study, but preliminary data suggest GASP cells can also be detected by our method. We therefore suggest that the effects described in the current report can be attributed to phenotypic variations.

Although *E. coli *cultures divide homogenously throughout the exponential and stationary phases, they differentiate into subsets while recovering from stationary phase: some bacteria are able to start growing rapidly and some remain non-dividing (Figure 3). When does the homogenous population give rise to these subsets of cells? Does it happen during stasis or upon transfer into the fresh growth medium? What could be the cause of this differentiation? The simplest explanation is that the non-dividing subset consists of dead or dying cells. However, this does not seem to be the case, since neither the dividing nor the non-dividing subpopulation contains significant numbers of cells that have lost membrane integrity as revealed by PI staining (Figure 4). It has been shown that stationary phase cells that can no longer be cultured have suffered more severe oxidative damage, which also results in higher expression in some of the stress regulons [33]. Thus, increased levels of expression of these stress response genes could serve as markers of oxidative damage and loss of ability to resume growth. On the other hand, the situation could be the other way around; the non-dividing subset might not grow because it is defective in its stress response and cannot resist the harmful conditions in stationary phase. Studies of various important components of bacterial stress response pathways [25,26] reveal that they are all expressed in fairly homogeneous fashion in stationary phase populations (Figure 5). Thus, the delay in recovery from stationary phase that we observe cannot be explained by defects in the stress response. It has been reported previously that two subpopulation of cells can be separated from early stationary phase culture by density gradient centrifugation [3], indicating that heterogeneity of the culture develops before it can be detected at the level of cell division. In addition, heterogeneity has been detected in the stationary phase cultures by centrifuging cells in different density gradients [20,34] or following the copy number of the chromosome [35]. Unfortunately, it is currently not clear how these parameters influence the ability of the cells do divide upon dilution of the stationary phase culture.

It may be wrong to presume that the non-dividing *E. coli *cells are necessarily defective and moribund. Bacteria can live in environments where changes occur rapidly and are often exposed to sudden stresses, including antimicrobials produced by competing microorganisms. Thus, it would be beneficial for a bacterial population to contain both non-dividing cells and those that are ready for rapid growth. The latter ensure that the population density is recovered rapidly, but they are vulnerable to reoccurrence of the stressful event (for example antibiotic treatment). In this case the dormant cells can recover the population after the stressor is removed [36]. It is an interesting question whether the division of the stationary phase culture into subpopulations relies on a phenotypic switch or is caused by mutations. We have made up to five cycles of growth into the stationary phase followed by dilutions into a fresh medium. In case there is a genetic cause behind the two subpopulations we would increase the fraction of cells recovering rapidly. The results of the growth experiment (data not shown) did not support this hypothesis – the fraction of rapidly resuscitating cells does not change, indicating that a phenotypic switch is involved. We recognized that the *E. coli *laboratory strains have passed many cycles involving growth to the stationary (an overnight culture) followed by dilution into a fresh medium. This process could select for mutations changing the relative fraction of the rapidly reviving subpopulation. We isolated a fresh *E. coli *strain from an environmental water sample, grow to the stationary phase and diluted into a fresh medium. The fresh isolate had a similar fraction of cells recovering rapidly from the stationary phase as the laboratory strains (data not shown) indicating the lack of selection pressure that would change the behaviour of the phenotypic switch in the typical laboratory growth conditions.

Most currently available antibiotics can only act on actively growing cells and have no effect on non-dividing bacteria. Indeed, when we added ampicillin to the culture while it was recovering from stationary phase, the subpopulation that had already started dividing lysed completely, whereas the non-dividing cells remained intact (Figure 6). Some of the bacteria that were sorted from the non-dividing subset after ampicillin treatment started growing when inoculated into fresh medium (Figure 7). These cells clearly fulfil the criteria of persisters as bacteria able to survive the antibiotic treatment and to resume growth after removal of the drug. The number of persisters in a culture, and the culturability of bacteria in general, are normally estimated by plating and counting the CFU. Plating results suggest that the subpopulation of persisters in a growing culture of wild type *E. coli *is too small for direct observation. Several attempts at surveillance and isolation of persisters have been carried out with *hip *(high persistence) mutants of *E. coli *[6,11,37,38]. In our experiments, we were able to assert that persisters in a wild type *E. coli *strain stem from a subset of stationary cells that remain non-dividing after transfer to growth medium. We calculated the minimal number of cells fulfilling the persister definition by extrapolating the growth curves in Figure 7 back to the time point 0. Assuming, quite conservatively, that persisters start to grow immediately after addition of fresh medium and with maximum speed, they constitute approximately 0.2% of the non-dividing cells. If their growth resumes later or more slowly, the number of persisters is even greater. Based on CFU counts, the persister frequency at the moment of inoculation is approximately 10^-4 ^and after three hours of growth (when the number of growing cells has increased about 10 times) it is dropping below 10^-5 ^[39]. Our results predict persister frequencies that are more than 10 fold higher The differences between previous estimations and our experiments might be due to loss of viability during plating, the procedure used in previous reports. This hypothesis has to be studied further. Also, we observe that most of the non-growing subpopulation remains non-dividing until these cells can be followed within a culture that is becoming increasingly crowded with growing bacteria. We do not know what would happen to these bacteria later, or on what their growth ability depends – if it is not completely lost.

The data relating persisters to a specific transcription profile were obtained from bacteria isolated on the basis of ampicillin tolerance [6] or translational inactivity (assessed by GFP expression) [38]. In these studies, this aspect of diversity was not considered and all non-growing cells were regarded as persisters. To address this problem, the kinetics of wake-up as well as the switch mechanisms and changes in bacterial cells that occur upon restart of growth must be studied further.

Although mutations accumulate in an *E. coli *culture initiated from a single colony the mutation rate could be too low for adaptation to the rapidly changing environment [40]. Thus, it would make sense in evolutionary terms to have a "back-up" of stress-resistant cells in this genetically homogeneous population that would be able to outlive the unfavourable conditions and found a new population if environmental conditions improve.

The benefit of switching between persister state and growing state is supported by theory and modelling. The idea that greater diversity is generally beneficial to a community in a fluctuating environment is called the insurance hypothesis and was at first intuitive [41]. This hypothesis has been tested by model building at different levels of complexity: both the effects of biodiversity (species richness) on ecosystem productivity [41] and diversity (genetic as well as phenotypic) on maintenance of a microbial population have been assessed [36,42]. These models provide strong theoretical support for the insurance hypothesis. A model of population growth has been developed that considers switching between cellular states in a dynamic environment that is sensed in a possibly noisy way by cellular sensors. Under such conditions, game theoretical analysis was applied to derive the evolutionarily stable strategy (ESS). Diversification was the optimal strategy when transitions between different selective environments could not be sensed [42].

## Conclusion

The current work describes a flow cytometry based simple approach for studying division of bacteria at single cell level. We conclude that *E. coli *cells behave in a uniform manner in the exponential and stationary growth phases. On the other hand, heterogeneity is observed after dilution of the stationary phase cells into a fresh medium. In addition, the method allows analyzing and isolating cells that persist treatment with bactericidal antibiotics. In principle, a variety of applications for the developed approach can be foreseen.

## Methods

### Bacterial strains and plasmids

All experiments were performed with strains derived from *E. coli *K-12 strain MG1655 (F-λ-*ilvG*-*rfb*-50 *rph*-1) [43]. Cloning was performed in *E. coli *strain DH5α [*supE44 ΔlacU169 (*Φ80 *lacZ *ΔM15) *hsdR17 recA1 endA1 gyrA96 thi-1 relA1*] [44].

Plasmid pMSLuxR (Additional file 4) was derived from plasmid pMS201 (gift from U. Alon) so that expression of the reporter gene *gfpmut2 *[15] is under the control of the *Vibrio fischeri luxICDABEG *operon promoter (gi: 297488, nucleotides 910–971). This promoter is regulated by the LuxR activator (gi: 5726577, nucleotides 214–1051), which is also encoded in the pMSLuxR plasmid (Km^R^). The LuxR regulator activates transcription is response to the presence of homoserine lactone (HSL) in the growth medium.

Plasmid pRpoSmut2 (Cm^R^) (Additional file 5) was derived from plasmid pACYC184 [45] and plasmids pJBdps (Additional file 6), pJBgadA, pJBkatE or pJBosmE (all Tet^R^) were derived from plasmid pJB866 [46] by fusing the regulator areas (regions upstream of the initiator codon) of *rpoS*, *dps*, *gadA*, *katE *or *osmE *to the ORF of the *gfpmut2 *reporter gene. Primers used for amplification of regulator sequences are listed in Table 1.

**Table 1:** Primers

Name	Sequence
*dpsF*	5'AATCTAGACTCGCTACTTTTCCTCTACACC3'
*dpsR*	5'AACATATGTTCATATCCTCTTGATGTTATGTCC3'
*gadAF*	5'AATCTAGATTTGATCGCCCGAACAGCAATG3'
*gadAR*	5'AACATATGGAACTCCTTAAATTTATTTGAAGGC3'
*katEF*	5'AATCTAGACTGTAGTTTAGCCGATTTAGCC3'
*katER*	5'AACATATGACTCGTCTCCTTAATTTATTACTG3'
*osmEF*	5'AATCTAGACCTTAAAGCTAACCCGTTGCTACTG3'
*osmER*	5'AACATATGCCGTCCTCTTGTTTATCAGCGTGTTAG3'
*rpoSF*	5'AAAAGCTTGCGCAACAATATTCAGGCACCATACG3'
*rpoSR*	5'AACATATGAGGTGGCTCCTACCCGTGATCCC3'

### Media and growth conditions

Cells were grown aerobically at 37°C in LB medium or on LB agar plates (Becton, Dickinson and Company). Antibiotics were used at the following concentrations when needed: kanamycin 50 μg/ml, tetracycline 15 μg/ml and chloramphenicol 10 μg/ml.

### GFP dilution experiments

For all experiments, an overnight culture of pMSLuxR containing MG1655 was diluted into 2 ml of fresh medium to optical density 0.05 (A600 nm). The culture was grown until the optical density reached 0.6 and expression of the reporter gene *gfpmut2 *was induced by addition of HSL. HSL was extracted with ethyl acetate from the supernatant of an *Erwinia carotovora subspecies carotovora *culture [47] or obtained from Sigma (N-(3-Oxooctanoyl)-L-homoserine lactone) and used at concentration 0.2 μM.

To follow cell division during exponential growth phase, the HSL-supplemented culture was diluted after 1 hour of HSL induction in 2 ml of fresh medium (without HSL) to optical density 0.05. The bacteria were further incubated; samples were taken every 20 or 30 minutes and analyzed by flow cytometry.

To follow cell division upon entry into stationary phase, the HSL-supplemented culture was grown until the optical density reached 2, then the cells were collected by centrifugation, washed with 1 × PBS and suspended in conditioned medium. The conditioned medium was obtained from a culture grown in the same conditions but without HSL. Cells were collected by centrifugation and the supernatant was filtered through a 0.2 μm filter. The bacteria were further incubated; samples were taken every 30 minutes and analyzed by flow cytometry.

To follow cell division in the stationary phase, the HSL-supplemented culture was grown until the optical density reached 3 (A600 nm), then the cells were treated as described for the "entry into stationary phase" experiment and incubated in conditioned medium without HSL for two days. Samples were taken at time points indicated and analyzed by flow cytometry.

To follow the division of bacteria recovering from stationary phase, the HSL-supplemented culture was grown to stationary phase and kept in that phase for 24 hours. The start of stationary phase was defined by the time when the optical density of the culture did not increase more than 5% over a 30 minute period. The stationary phase culture was diluted into 2 ml of fresh medium (without HSL) to optical density 0.05 (A600 nm). The bacteria were further incubated; samples were taken every 30 minutes and analyzed by flow cytometry.

### Staining with propidium iodide

Cells were taken from the GFP dilution experiment, a culture that had recovered for 2 hours from stationary phase. They were washed twice with cold 1 × PBS and divided into two halves, one of which was left untreated while the other was stained at room temperature for 5 minutes with 10 μg/ml propidium iodide in 1 × PBS. The cells were immediately analysed by flow cytometry.

### Expression from stationary phase-specific promoters

Overnight stationary-phase cultures of MG1655 containing plasmids pRpoSmut2, pJBdps, pJBgadA, pJBkatE or pJBosmE were diluted into 2 ml of fresh medium to optical density 0.05 (A600 nm). The bacteria were further incubated, samples were taken at inidicated times and analyzed by flow cytometry.

### Flow cytometry

Flow cytometry was performed using a FacsCalibur or LSR II (Becton Dickinson and Company) with a laser beam maximum at 488 nm. At least 30,000 events per sample were counted. The results were analyzed by FlowJo 7.2.1. software (Treestar, Inc.). Samples of bacterial cultures were mixed 1:1 with 1 × PBS supplemented with 30% glycerol and stored at -80°C until analysis (unless otherwise stated). Samples thus preserved do not differ from fresh samples in any way on flow cytometric analysis.

### Treatment with ampicillin

MG1655 cultures containing pMSLuxR were supplemented with HSL as in all the dilution experiments, grown to stationary phase and incubated in that phase for 24 hours. The stationary phase culture was diluted into 20 ml of fresh medium to optical density 0.02 (A 600 nm). Recovery from stationary phase was monitored by taking samples every 30 minutes. After 2 hours of incubation in fresh medium, ampicillin was added to a concentration of 200 μg/ml, the culture was incubated further, samples were collected and analyzed by flow cytometry.

For resuscitation, 1,000,000 fluorescent particles and 1,000,000 non-fluorescent particles (as a negative control) were collected by flow sorting (using FacsAria, Becton Dickinson and Company) 30, 60, 90 and 180 minutes after addition of ampicillin. The negative control was collected from a sample that had been incubated with ampicillin for 180 minutes. Bacteria were collected in 1 × PBS. The collected cells were mixed 1:1 with 2 × LB medium containing no NaCl to produce a medium with ionic strength equal to LB. Cells were incubated for up to 6 hours on a shaker at 37°C, and samples were taken and analyzed by flow cytometry.

### Reproducibility of experiments

All experiments described in the current manuscript have been repeated at least for three times. Typical experiments are presented.

## Authors' contributions

AJ, TT and NK conceived the study. JR, AJ and HL collected the experimental data. JR, TT and NK wrote the manuscript. All authors read and approved the final manuscript.

## Supplementary Material

Additional file 1 The GFP dilution experiment shown in Figure 2(C) performed with an IPTG inducible system. Click here for file

Additional file 2 Negative control for expression of stationary phase-specific genes (Figure 5). Click here for file

Additional file 3 Quantification of cell lysis with ampicillin (Figure 6). Click here for file

Additional file 4 Sequence of plasmid pMSLuxR. Click here for file

Additional file 5 Sequence of plasmid pRpoSmut2. Click here for file

Additional file 6 Sequence of plasmid pJBdps. Click here for file

## References

[B1] Smits WK, Kuipers OP, Veening JW (2006). Phenotypic variation in bacteria: the role of feedback regulation. Nat Rev Microbiol.

[B2] Dubnau D, Losick R (2006). Bistability in bacteria. Mol Microbiol.

[B3] Cuny C, Dukan L, Fraysse L, Ballesteros M, Dukan S (2005). Investigation of the first events leading to loss of culturability during Escherichia coli starvation: future nonculturable bacteria form a subpopulation. J Bacteriol.

[B4] Raser JM, O'Shea EK (2005). Noise in gene expression: origins, consequences, and control. Science.

[B5] Lewis K (2007). Persister cells, dormancy and infectious disease. Nat Rev Microbiol.

[B6] Keren I, Shah D, Spoering A, Kaldalu N, Lewis K (2004). Specialized persister cells and the mechanism of multidrug tolerance in Escherichia coli. J Bacteriol.

[B7] Spoering AL, Lewis K (2001). Biofilms and planktonic cells of Pseudomonas aeruginosa have similar resistance to killing by antimicrobials. J Bacteriol.

[B8] Nystrom T (2003). Conditional senescence in bacteria: death of the immortals. Mol Microbiol.

[B9] Oliver JD (2005). The viable but nonculturable state in bacteria. J Microbiol.

[B10] Nystrom T (2001). Not quite dead enough: on bacterial life, culturability, senescence, and death. Arch Microbiol.

[B11] Balaban NQ, Merrin J, Chait R, Kowalik L, Leibler S (2004). Bacterial persistence as a phenotypic switch. Science.

[B12] Stewart EJ, Madden R, Paul G, Taddei F (2005). Aging and death in an organism that reproduces by morphologically symmetric division. PLoS Biol.

[B13] Ahmer BM (2004). Cell-to-cell signalling in Escherichia coli and Salmonella enterica. Mol Microbiol.

[B14] Vimberg V, Tats A, Remm M, Tenson T (2007). Translation initiation region sequence preferences in Escherichia coli. BMC Mol Biol.

[B15] Cormack BP, Valdivia RH, Falkow S (1996). FACS-optimized mutants of the green fluorescent protein (GFP). Gene.

[B16] van den Bogaart G, Hermans N, Krasnikov V, Poolman B (2007). Protein mobility and diffusive barriers in Escherichia coli: consequences of osmotic stress. Mol Microbiol.

[B17] Elowitz MB, Surette MG, Wolf PE, Stock JB, Leibler S (1999). Protein mobility in the cytoplasm of Escherichia coli. J Bacteriol.

[B18] Mullineaux CW, Nenninger A, Ray N, Robinson C (2006). Diffusion of green fluorescent protein in three cell environments in Escherichia coli. J Bacteriol.

[B19] Unge A, Tombolini R, Molbak L, Jansson JK (1999). Simultaneous monitoring of cell number and metabolic activity of specific bacterial populations with a dual gfp-luxAB marker system. Appl Environ Microbiol.

[B20] Siegele DA, Alminor M, Kolter R, Kjelleberg S (1993). Approaches to the Study of Survival and Death in Stationary Phase Escherichia Coli.. Starvation in Bacteria.

[B21] Bouvier T, Troussellier M, Anzil A, Courties C, Servais P (2001). Using light scatter signal to estimate bacterial biovolume by flow cytometry. Cytometry.

[B22] Julia O, Comas J, Vives-Rego J (2000). Second-order functions are the simplest correlations between flow cytometric light scatter and bacterial diameter. J Microbiol Methods.

[B23] Shapiro HM (2003). Practical Flow Cytometry, Fourth Edition. http://eu.wiley.com/WileyCDA/WileyTitle/productCd-0471411256.html.

[B24] Caron GN, Stephens P, Badley RA (1998). Assessment of bacterial viability status by flow cytometry and single cell sorting. J Appl Microbiol.

[B25] Shimada T, Makinoshima H, Ogawa Y, Miki T, Maeda M, Ishihama A (2004). Classification and strength measurement of stationary-phase promoters by use of a newly developed promoter cloning vector. J Bacteriol.

[B26] Weber H, Polen T, Heuveling J, Wendisch VF, Hengge R (2005). Genome-wide analysis of the general stress response network in Escherichia coli: sigmaS-dependent genes, promoters, and sigma factor selectivity. J Bacteriol.

[B27] Levin BR, Rozen DE (2006). Non-inherited antibiotic resistance. Nat Rev Microbiol.

[B28] Wagner JK, Brun YV (2007). Out on a limb: how the Caulobacter stalk can boost the study of bacterial cell shape. Mol Microbiol.

[B29] Kearns DB, Losick R (2005). Cell population heterogeneity during growth of Bacillus subtilis. Genes Dev.

[B30] Bjedov I, Tenaillon O, Gerard B, Souza V, Denamur E, Radman M, Taddei F, Matic I (2003). Stress-induced mutagenesis in bacteria. Science.

[B31] Finkel SE, Kolter R (1999). Evolution of microbial diversity during prolonged starvation. Proc Natl Acad Sci U S A.

[B32] Zambrano MM, Siegele DA, Almiron M, Tormo A, Kolter R (1993). Microbial competition: Escherichia coli mutants that take over stationary phase cultures. Science.

[B33] Desnues B, Cuny C, Gregori G, Dukan S, Aguilaniu H, Nystrom T (2003). Differential oxidative damage and expression of stress defence regulons in culturable and non-culturable Escherichia coli cells. EMBO Rep.

[B34] Makinoshima H, Nishimura A, Ishihama A (2002). Fractionation of Escherichia coli cell populations at different stages during growth transition to stationary phase. Mol Microbiol.

[B35] Akerlund T, Nordstrom K, Bernander R (1995). Analysis of cell size and DNA content in exponentially growing and stationary-phase batch cultures of Escherichia coli. J Bacteriol.

[B36] Kussell E, Kishony R, Balaban NQ, Leibler S (2005). Bacterial persistence: a model of survival in changing environments. Genetics.

[B37] Moyed HS, Bertrand KP (1983). hipA, a newly recognized gene of Escherichia coli K-12 that affects frequency of persistence after inhibition of murein synthesis. J Bacteriol.

[B38] Shah D, Zhang Z, Khodursky A, Kaldalu N, Kurg K, Lewis K (2006). Persisters: a distinct physiological state of E. coli. BMC Microbiol.

[B39] Keren I, Kaldalu N, Spoering A, Wang Y, Lewis K (2004). Persister cells and tolerance to antimicrobials. FEMS Microbiol Lett.

[B40] Drake JW (1991). A constant rate of spontaneous mutation in DNA-based microbes. Proc Natl Acad Sci U S A.

[B41] Yachi S, Loreau M (1999). Biodiversity and ecosystem productivity in a fluctuating environment: the insurance hypothesis. Proc Natl Acad Sci U S A.

[B42] Wolf DM, Vazirani VV, Arkin AP (2005). Diversity in times of adversity: probabilistic strategies in microbial survival games. J Theor Biol.

[B43] Blattner FR, Plunkett G, Bloch CA, Perna NT, Burland V, Riley M, Collado-Vides J, Glasner JD, Rode CK, Mayhew GF, Gregor J, Davis NW, Kirkpatrick HA, Goeden MA, Rose DJ, Mau B, Shao Y (1997). The complete genome sequence of Escherichia coli K-12. Science.

[B44] Sambrook J, Russell DW (2001). Molecular cloning : a laboratory manual.

[B45] Rose RE (1988). The nucleotide sequence of pACYC184. Nucleic Acids Res.

[B46] Blatny JM, Brautaset T, Winther-Larsen HC, Haugan K, Valla S (1997). Construction and use of a versatile set of broad-host-range cloning and expression vectors based on the RK2 replicon. Appl Environ Microbiol.

[B47] Koiv V, Mae A (2001). Quorum sensing controls the synthesis of virulence factors by modulating rsmA gene expression in Erwinia carotovora subsp. carotovora. Mol Genet Genomics.

