# Supplementary figures and images for: Cell division in Escherichia coli cultures monitored at single cell resolution
Source: BMC Microbiol. 2008 Apr 23;8:68. doi: 10.1186/1471-2180-8-68 (PMC2377270; doi:10.1186/1471-2180-8-68)

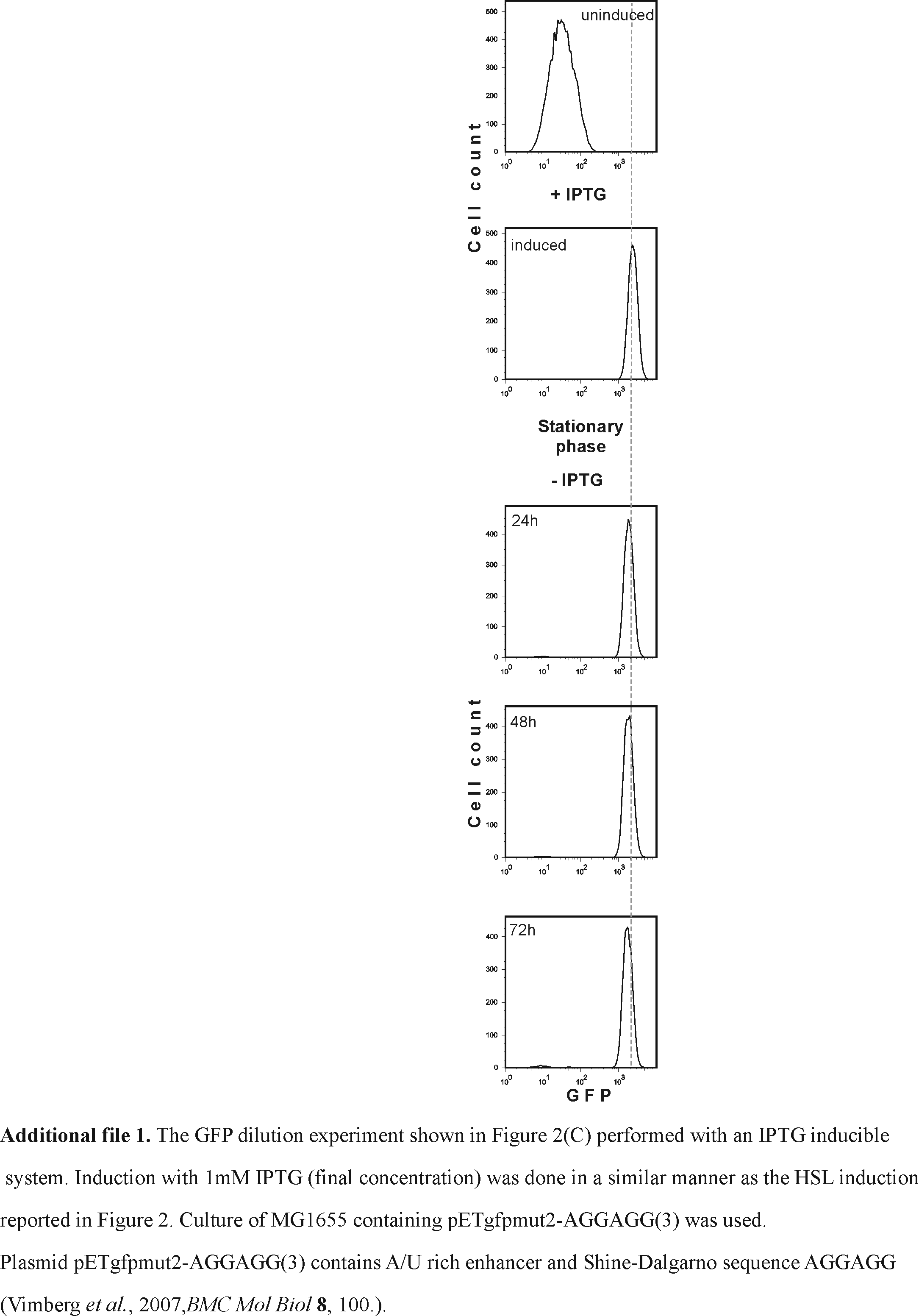

Supplement: Additional file 1 — The GFP dilution experiment shown in Figure 2(C) performed with an IPTG inducible system. [file 1471-2180-8-68-S1.tiff]

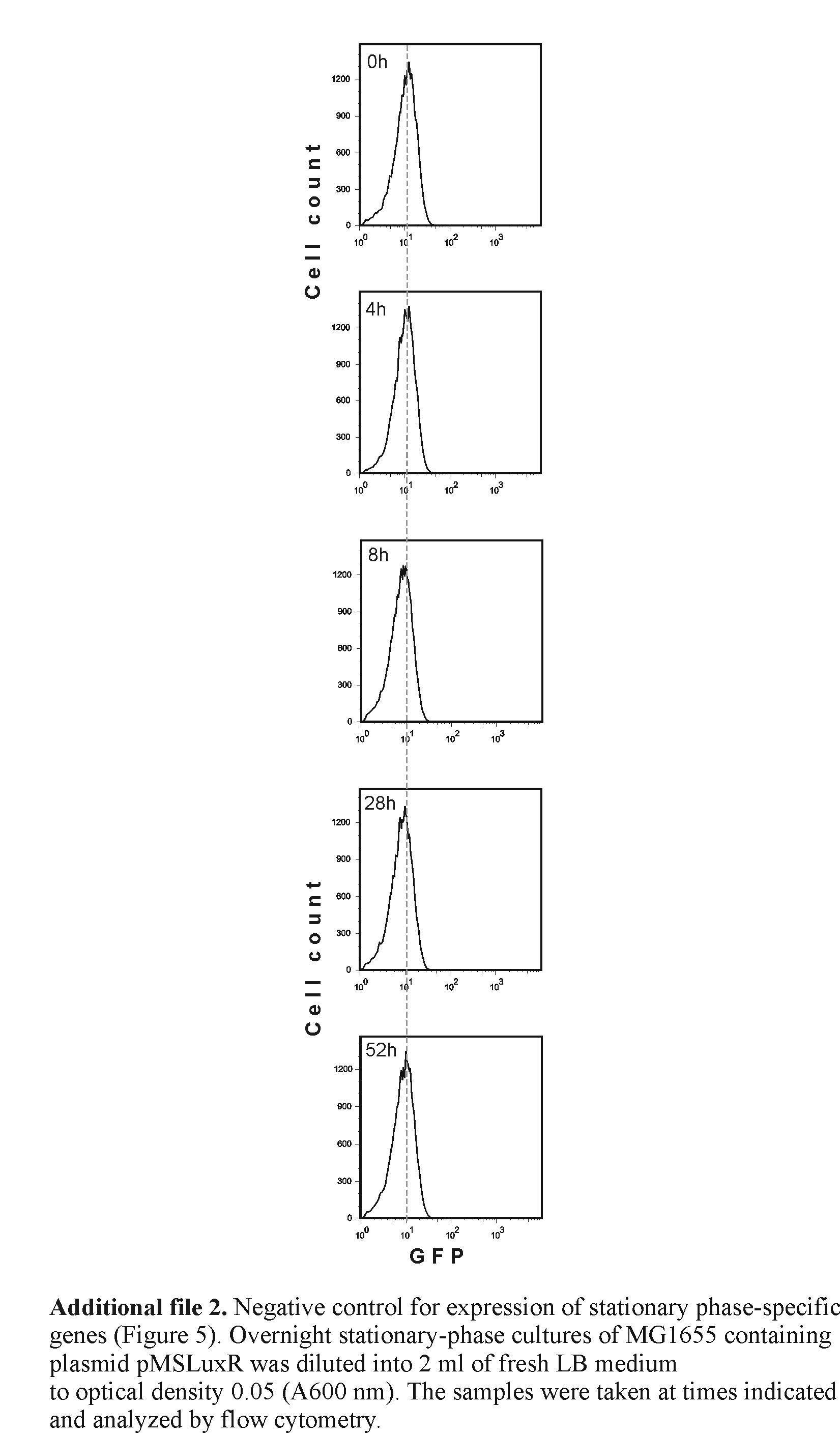

Supplement: Additional file 2 — Negative control for expression of stationary phase-specific genes (Figure 5). [file 1471-2180-8-68-S2.tiff]

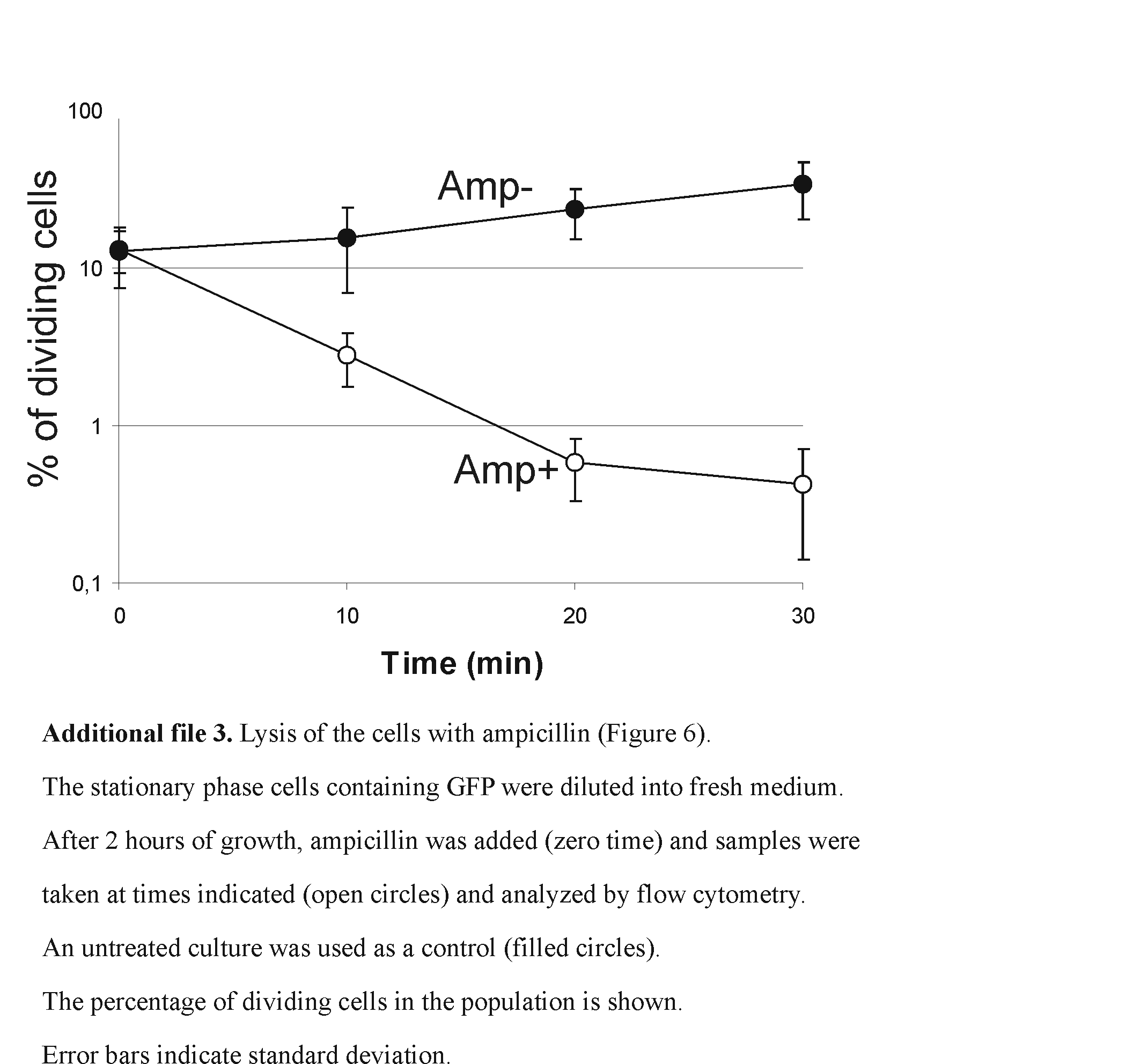

Supplement: Additional file 3 — Quantification of cell lysis with ampicillin (Figure 6). [file 1471-2180-8-68-S3.tiff]
